# A Novel Ecdysone Receptor Mediates Steroid-Regulated Developmental Events during the Mid-Third Instar of *Drosophila*

**DOI:** 10.1371/journal.pgen.1000102

**Published:** 2008-06-20

**Authors:** Benjamin F. B. Costantino, Daniel K. Bricker, Kelly Alexandre, Kate Shen, John R. Merriam, Christophe Antoniewski, Jenna L. Callender, Vincent C. Henrich, Asaf Presente, Andrew J. Andres

**Affiliations:** 1 School of Life Sciences, University of Nevada Las Vegas, Las Vegas, Nevada, United States of America; 2 Department of Molecular, Cell and Developmental Biology, University of California Los Angeles, Los Angeles, California, United States of America; 3 Department of Developmental Biology, Institut Pasteur, Paris, France; 4 Center for Biotechnology, Genomics, and Health Research, University of North Carolina Greensboro, Greensboro, North Carolina, United States of America; University of California San Francisco, United States of America

## Abstract

The larval salivary gland of *Drosophila melanogaster* synthesizes and secretes glue glycoproteins that cement developing animals to a solid surface during metamorphosis. The steroid hormone 20-hydroxyecdysone (20E) is an essential signaling molecule that modulates most of the physiological functions of the larval gland. At the end of larval development, it is known that 20E—signaling through a nuclear receptor heterodimer consisting of EcR and USP—induces the early and late puffing cascade of the polytene chromosomes and causes the exocytosis of stored glue granules into the lumen of the gland. It has also been reported that an earlier pulse of hormone induces the temporally and spatially specific transcriptional activation of the glue genes; however, the receptor responsible for triggering this response has not been characterized. Here we show that the coordinated expression of the glue genes midway through the third instar is mediated by 20E acting to induce genes of the *Broad Complex (BRC)* through a receptor that is not an EcR/USP heterodimer. This result is novel because it demonstrates for the first time that at least some 20E-mediated, mid-larval, developmental responses are controlled by an uncharacterized receptor that does not contain an RXR-like component.

## Author Summary

During animal development the physiological response of individual tissues is often “reprogrammed” in response to signaling molecules. One important example is the activity of nuclear-hormone receptors that are controlled by small lipid compounds such as steroids and retinoids. Thus, understanding how tissue-specific developmental and physiological responses are regulated by these systemic ligands is a fundamental question of cell biology. *Drosophila* is an important model system in which to investigate this question because of its 100-year history of analyzing mutants that affect complex biological processes, and because researchers possess a powerful “toolkit” that allows for precise tissue- and temporal-specific expression and silencing of almost any gene in the genome. Furthermore, during the metamorphosis of *Drosophila*, the body plan is completely reorganized from that of a larva (specialized for growth and feeding) to that of an imago (specialized for reproduction and dispersal) by a single steroid hormone. Here we examine the molecular events that control different physiological responses within a single target tissue to different pulses of 20E. We show for the first time that these temporally specific events within the same tissue are controlled in part by different 20E receptors.

## Introduction

During metamorphosis in *Drosophila melanogaster*, pulses of the 20E steroid hormone, stimulate diverse tissue-specific responses such as the histolysis of many larval tissues and the simultaneous differentiation of adult structures from imaginal discs [reviewed in 1]. In addition, multiple pulses of 20E that occur during the last larval instar (L3) trigger different responses within the same target tissue, raising the interesting question of how a generalized developmental signal is manifested into distinct physiological responses that are separated by time. The larval/prepupal salivary gland is an ideal assay system in which to investigate the molecular mechanisms responsible for such temporally specific developmental specifications. In a 36-hour period, the gland responds to three distinct pulses of 20E in three fundamentally different ways.

During most of larval life, the salivary gland is engaged in the synthesis of non-digestive enzymes that most likely aid in the lubrication of the food through the gut [2]–[4]. However, about midway through the L3 stage, the pattern of gene expression is altered dramatically by the synchronous activation of a small number of genes (∼8) that are abundantly expressed in the salivary gland [5]. These are known to encode components of the glue mix that cements animals to a solid surface during metamorphosis, and they were first identified because their induction is responsible for the “intermolt” puffs formed on the giant polytene chromosomes of the gland [6],[7].

Approximately 18 hours later, in response to the pulse of 20E that occurs at the end of L3, glue synthesis abruptly ceases [5],[8] because the hormone represses transcription from these genes [9],[10]. At this time, the salivary gland begins to express another set of genes, many of which were originally described because they formed “early” and “late” puffs on the polytene chromosomes [reviewed in 11]. The end result of this 20E-mediated response is that glue granules are secreted into the lumen of the gland [12],[13].

Finally at the end of prepupal development 10–12 hours later, the salivary gland responds to yet another pulse of 20E to initiate the programmed cell death of the tissue via a pathway that involves components of both autophagy and caspase activation [14],[15].

The details of how 20E initiates glue secretion and gland histolysis are well understood. The hormone is known to bind to a receptor consisting of a heterodimer of EcR (FBgn0000546) and USP (FBgn0003964) proteins [16]–[18]. Both receptor components are members of the nuclear-hormone receptor superfamily, both contain well conserved DNA- and ligand-binding domains, and both are needed for the physiological responses of target tissues to 20E at these times [reviewed in 19]. However, little is known concerning the mechanism of receptor mediation during the middle of L3 when glue genes are coordinately activated. Although it is generally assumed that these events are also mediated by a receptor consisting of EcR and USP, other explanations can be invoked including the use of a different 20E receptor.

Here we examine the requirements of EcR and USP for the induction of the glue genes at mid L3. By employing the GAL4/UAS binary expression system [20] with transgenic inducible dominant-negative and RNAi constructs, we are able to limit perturbations of 20E signaling specifically to the salivary gland at defined developmental stages. We show that 20E is responsible for inducing a tagged glue transgene as a secondary response to the hormone, and that the 20E-inducible primary-response genes of the *Broad Complex (BRC)* (FBgn0000210) are sufficient to initiate this programmed developmental response. However, we clearly demonstrate that the mid-instar hormone response requires a receptor that has not yet been characterized. The receptor consists of EcR but not USP. These results challenge the traditional model that most developmental events triggered by 20E must signal through a heterodimer of EcR and USP, and they support an alternative explanation in which either EcR homodimers or other members of the nuclear-hormone receptor superfamily play an active role in the diversity of responses to 20E during *Drosophila* development.

## Results

### An *Sgs3* Transgene Is Induced by 20E

It is generally assumed that the glue genes [*Sgs1* (FBgn0003372), *Sgs3* (FBgn0003373), *Sgs4* (FBgn0003374), *Sgs5* (FBgn0003375), *Sgs6* (FBgn0003376), *Sgs7* (FBgn0003377), *Sgs8* (FBgn0003378), and *I71-7* (FBgn0004592)] are induced by a pulse of 20E that occurs midway through the third instar. This inference is based on the dramatically coordinated developmental induction at mid L3 of most of these genes [5], and on studies in which *Sgs* expression is examined in backgrounds mutant for genes thought to be involved in 20E production or transport [21],[22]. The model further proposes that induction of the glue genes occurs as a secondary response to 20E because *Sgs* expression is significantly perturbed in mutants defective for *BRC* and *E74* (FBgn0000567), which are known to be direct targets for the hormone/receptor complex [23]–[25]. However, an *Sgs3*-derived reporter transgene is induced when temperature-sensitive *ecd1^ts^* (FBgn0000543) mutants—known to produce low circulating levels of 20E [26]—are shifted to the non-permissive temperature before the L3 stage, and the same GFP reporter is also induced in animals that are mutant for USP, EcR-B1, and EcR-B2 receptor components [13].

Thus, the literature contains contradictory reports concerning the role of 20E in inducing the glue genes. Therefore, we began this analysis with an *in-vitro* culture of salivary glands dissected from mid L3 because we are not aware of any published reports that directly test if glue-gene transcription can be induced by 20E in glands cultured from wildtype animals. To simplify the analysis, we dissected salivary glands from a line of flies in which the coding information for *Sgs3* had been tagged with *GFP* (*glueGRN*) (FBst0005884). This stock (previously called *SgsGFP*) contains adequate regulatory information for the proper temporal, spatial, and high-level expression of the *Sgs3* gene. It has also been extensively characterized and shown to be an accurate reporter for the secretion and expectoration of endogenous SGS3 glue protein [13].

**Figure 1:**
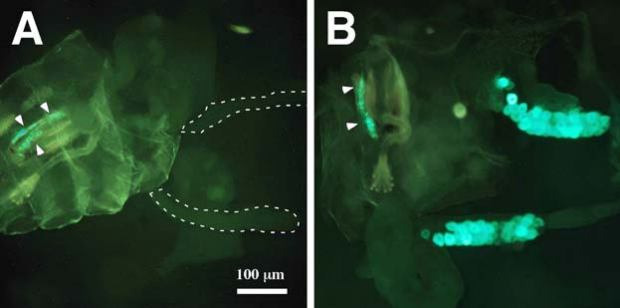
Glue Genes are Induced by 20E in Cultured Glands. Mid-L3 animals were torn in half and incubated with ethanol as a control (A), or with 20E at a final concentration of 10^−8^ M (B). The induction of glue proteins in the salivary glands was detected by the expression of a *GFP*-tagged *Sgs3* gene (*glueGRN*). Note that the positions of the salivary glands in (A) are outlined with dashed lines. The fluorescence detected in the pharynx (arrowheads) is non-specific and was used to standardize photographic exposures. Both photographs were taken at the same magnification indicated by the bar in A.

Larvae were synchronized at hatching and raised to the early-L3 stage approximately 4–5 hours prior to the normal transcriptional induction of the glue genes. Salivary glands were then dissected and exposed to media containing different concentrations of 20E (ranging 10^−9^ to 10^−6^ M) or in medium without hormone. Under these circumstances glueGRN accumulation was detected 4–6 hours later in cultures incubated with low 20E concentrations (Figure 1), but not in untreated cultures or those incubated with higher concentrations of the hormone. It should be noted that this response is not robust because only ∼30% of the dissected glands produce glueGRN when treated. Presumably the results are variable because the culture conditions have not been optimized and/or the animals are not staged in a precise enough manner. However, it is significant to note that the only cultures in which glueGRN was detected were those incubated in either 10^−8^ M (8 out of 20) or 10^−9^ M (4 out of 20). Thus, the concentration needed for production of glueGRN is 2–3 orders of magnitude lower than the titer (∼10^−6^ M) reported to trigger “early” polytene puff formation, imaginal disc eversion, and glue secretion [13],[27],[28]—all developmental events that occur near puparium formation in response to a much better characterized pulse of 20E. The result is consistent with the concentration of a small pulse of hormone that has been reported to occur in the hemolymph of developing larvae a few hours prior to the transcriptional activation of the glue genes [29].

### *Sgs3*-Derived Transgenes Require Functional EcR for Expression

The *EcR* gene encodes three different protein isoforms, EcR-A, EcR-B1, and EcR-B2. All three contain the same DNA- and ligand-binding domains, but they contain different amino terminal A/B sequences due to the use of alternative promoters and differential splicing [16]. Null mutations for *EcR* die early in development and cannot be assayed for glue synthesis [30]. However, mutations that remove EcR-B1 and EcR-B2 [31] do produce glue [13]. These observations raise the possibility that either EcR is not required for the induction of *Sgs3*, or that any EcR isoform is sufficient for the process. To distinguish between these possibilities and to take advantage of more powerful genetic tools that allow for tissue-specific manipulations of gene products, we utilized the GAL4/UAS binary expression system [20] to analyze glue-gene induction in developing salivary glands.

**Figure 2:**
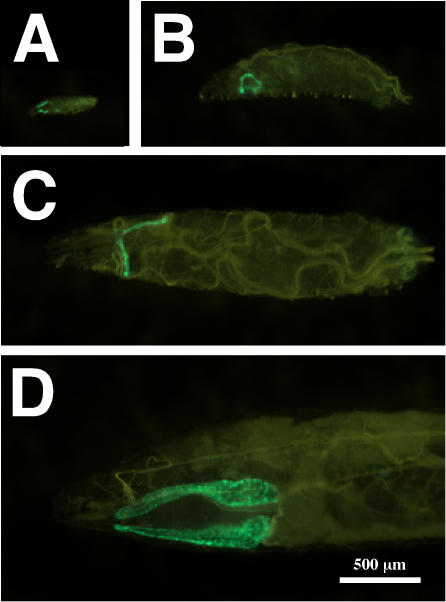
sgGal4, a *GawB*-Derived Driver, Has an Expression Pattern Restricted to the Larval Salivary Glands. Flies containing *sgGal4* were crossed to a *GFP* responder (*UAS-GFP.nls*) and all developmental stages were examined for the localization of GFP in the nuclei of live animals. The only tissues that contained green protein were the salivary glands. Depicted are a first-instar larva (A), a second-instar larva (B), an early-third-instar larva (C), and the anterior half of a late-third instar larva (D). Anterior is to the left in all photographs, and all were taken at the same magnification indicated by the bar in (D).

To perform this analysis in the most precise way, it was first necessary to identify a temporally and spatially restricted driver—a transgenic stock of flies in which the *Gal4* transcription factor is under the control of specific *Drosophila* enhancers that limit its expression to larval salivary glands at least 10 hours preceding the normal induction of *Sgs3*. In our search for the best reagent, we noticed that a number of the drivers classified as salivary-gland specific were produced from the *P{GawB}* enhancer-trap element (FBtp0000352). Our lab and others have observed that *GawB*-derived elements display constitutive expression of GAL4 in larval salivary glands [32], perhaps because part of the *GawB* vector contains a cryptic larval salivary-gland-specific enhancer element. To test this hypothesis we used a *hs-Gal4* stock that contains the *Hsp70Bb* (FBgn0013278) controlling elements driving *Gal4* in a *GawB* vector. In the absence of heat stress these animals produced GAL4 in the L1 (first instar), L2 (second instar), and L3 salivary glands as indicated when they were crossed to the *GFP.nls* responder. In this stock GFP is expressed under GAL4 control (it contains *UAS* elements that are binding sites for the GAL4 transcription factor) and it is targeted to nuclei (Figure 2). Thus, in subsequent experiments we used *hsGal4* (now referred to as *sgGal4*) to drive spatially restricted expression of *UAS*-transgenes only in larval salivary glands.

With a spatially restricted driver on hand, we now crossed *sgGal4* to both *UAS-dominant-negative- (EcR-DN)* and *UAS-RNA-interference- (EcRi)* constructs of *EcR*. The EcR-DN protein is defective in ligand-activated transactivation so that it competes with endogenous EcR isoforms to block normal hormone responses [33]. The *EcRi* construct contains an inverted repeat of a DNA region common to *EcR-A*, *B1*, and *B2* so that its expression silences all isoforms [34]. When either was crossed to a tester stock containing *sgGal4* and *glueGRN*, no green fluorescence was detected in L3 larval glands. These results are consistent with a requirement that at least one EcR isoform must be present in the salivary gland for glueGRN synthesis (data not shown).

**Figure 3:**
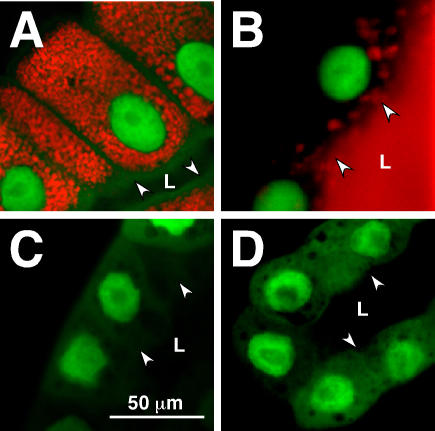
20E-Mediated glueRED Induction Requires Functional EcR. Confocal images of salivary glands from stocks containing *sgGal4*; *GFP.nls*; *glueRED* transgenes are presented. Normally glueRED is synthesized during the mid-L3 stage and loaded into large granules that remain within the cell until 6–8 hours prior to pupariation (A). At the end of the instar (in response to another pulse of 20E), glue becomes secreted into the lumen (B). No glueRED is produced in animals expressing the *EcR-DN* (C) or *EcRi* (D) transgenes. Note that *GFP.nls* is expressed in both types of EcR-compromised glands. It marks nuclei that are able to survive and polytenize to the same degree in all cells presented. All photos were taken at the same magnification indicated by the bar in (C). Arrowheads and “L” mark the position of the lumen into which glue should be secreted.

One potential caveat with the above experiments is that by perturbing EcR in the salivary gland, we were killing it or causing it to develop too slowly to induce the *Sgs3* transgene. To address this possibility, we utilized another tester stock containing three transgenic elements: *glueRED*; *GFP.nls*; *sgGal4*. The *glueRED* element is an endogenously tagged *Sgs3* gene (under its own promoter/enhancer elements). It contains the same DNA sequence as *glueGRN* except the coding information for *GFP* is replaced with that of *DsRED* [35]. As with *glueGRN*, the *glueRED* element produces a protein that is synthesized (Figure 3A), secreted (Figure 3B), and expectorated in exactly the same manner as endogenous SGS3. Thus, when this tester stock was crossed to *EcR-DN* (Figure 3C) or *EcRi* (Figure 3D) no glueRED was produced, but GFP is still localized to nuclei that are similar in size to those of the control glands producing glueRED (Figure 3A,B). These results indicate that neither *EcR-DN* nor *EcRi* expression is killing the cells or preventing their normal nuclear polytenization. Thus, EcR function in the salivary gland is required for glueGRN and glueRED production.

### *Sgs3* Transgenes Can Be Induced by Any Isoform of EcR

**Figure 4:**
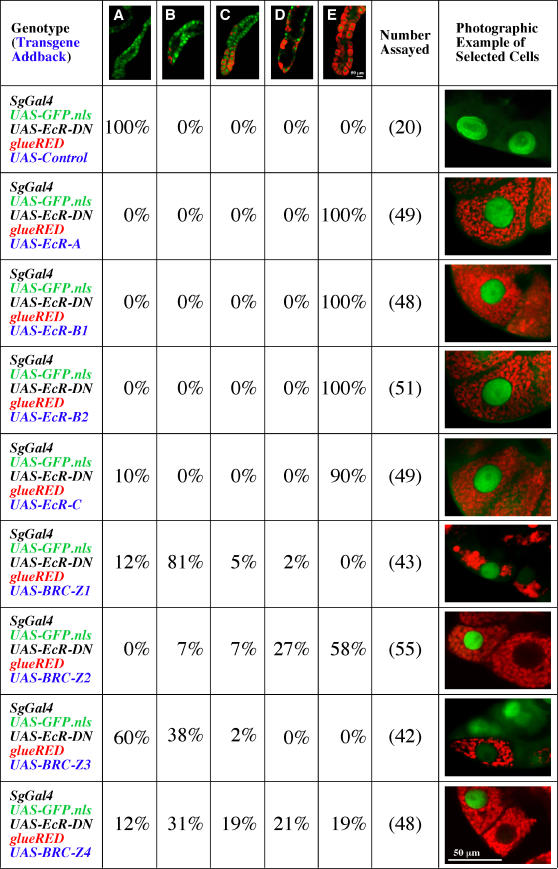
Any Isoform of EcR or BRC is Sufficient to Induce glueRED Synthesis. The percentage of glands displaying each synthesis phenotype is indicated below a low-resolution representative (all photographed at the same magnification as indicated in E). Categories include glands that are not producing any glueRED (A), glands in which only a few cells produce glueRED (B), glands in which approximately 25% of the cells are producing glueRED (C), glands in which approximately half the cells are producing glueRED (D), and glands in which all the cells are producing glueRED (E). Higher resolution images of cells representing each genotype (transgene addbacks) were all taken at the same magnification (indicated bottom right).

To test the hypothesis that any isoform of EcR can be used to induce glue synthesis, we crossed each *UAS-EcR* isoform-specific transgene into a background in which *EcR-DN* was expressed in the salivary gland (under *sgGal4* control) using the *glueRED* and *GFP.nls* transgenes to assay gland physiology. To confirm that extra copies of *UAS*-transgenes were not diluting the effects of *EcR-DN* in a non-specific manner, we included a *UAS-control* construct that contains a cassette of UAS/GAL4 binding sites. By itself, the expression of the *UAS-control* does not lead to a block in glueRED synthesis when driven by *sgGal4* (data not shown). Furthermore when crossed into an animal producing EcR-DN and GFP.nls, it does not overcome the block in glueRED synthesis (Figure 4). This control eliminates the concern that the expression of EcR-DN may be reduced by the introduction of an additional transgene containing *UAS* elements.

In contrast to the *UAS-control*, introducing each of the known EcR-specific isoforms into the same genetic background completely rescues the block in the production of glueRED caused by EcR-DN. The rescue is fully penetrant and normal in the semi-quantitative scoring scheme that is presented in Figure 4. It is even more interesting because an artificially constructed EcR isoform—EcR-C, which contains only the common-regions of EcR because it is missing the isoform-specific A/B domain—also rescues the block in glueRED synthesis in approximately 90% of the animals examined. These results confirm an earlier conclusion that any isoform of EcR expressed in the salivary gland is capable of transmitting the 20E signal to induce the transcription of *Sgs3*-derived genes.

### Proteins Encoded by the *Broad Complex (BRC)* Control the Expression of *Sgs3* Transgenes

The *BRC* is a large transcription unit that produces several different isoforms of a transcription factor containing C_2_H_2_ zinc-fingers. Although multiple transcripts are derived from the locus [36], only four general types of proteins are produced. Each isoform contains an identical NH_2_ terminus, but it has a different combination of DNA binding domains [37]. The four proteins, referred to as BRC-Z1, BRC-Z2, BRC-Z3, and BRC-Z4, have been shown to play an important role in the production of SGS3 and other glue proteins. This conclusion is based on the phenotypic analyses of null- or isoform-specific hypomorphic mutants that either do not produce SGS3 or display a prolonged developmental delay in the accumulation of transcripts from the locus [13],[23],[24],[38]. Because it has been shown that *BRC* is regulated as a primary response to 20E (the hormone/receptor complex directly binds to DNA elements within the gene and induction does not require *de novo* protein synthesis) [39], the above effects on *Sgs3* activation have led to a model in which glue production occurs as a secondary response to the hormone. Thus, the BRC zinc-finger transcription factors are probably responsible for activating promoter/enhancer elements within the glue genes as suggested by DNA binding studies on *Sgs4* [40].

To test this hypothesis in more detail, we utilized transgenic stocks in which each *BRC-Z* isoform was expressed under *UAS* control in larvae also containing *glueRED*; *sgGal4*; *GFP.nls*; and *EcR-DN*. As indicated in Figure 4, each BRC-Z isoform is capable of partially rescuing the block in glue synthesis imposed by the production of EcR-DN. Rescue was scored using five categories that indicated the approximate percentage of cells within a gland that produced glueRED (none, few, ∼25%, ∼50%, 100%). However, not all BRC isoforms are equal in their ability to suppress the synthesis defect imposed by EcR-DN. BRC-Z2 (no glands were observed that were completely empty of glue, and 58% had full wildtype levels) and BRC-Z4 rescue the best; whereas, BRC-Z1 and BRC-Z3 (60% of the animals have glands with no glueRED) rescue poorly. The variability in rescuing the synthesis-blocked phenotype may reflect the partially redundant activities or regulatory dependencies that have been reported among the four types of BRC isoforms [37], or it may reflect the differences in expression levels among the different transgenes.

Two additional points are worth noting. First, expression of all forms of *UAS-BRC* altered the expression/localization of GFP.nls in some cells, but this failure to localize GFP did not correlate with a defect in glueRED production. In all cases, a few cells producing glueRED were observed with large prominent nuclei that did not contain GFP. Because we never observe this effect in the experiments performed with EcR isoforms or the *UAS-control*, it is unlikely that extra transgenes containing *UAS* elements are titrating a limiting amount of GAL4 transcription factor.

Second, we sometimes observe the appearance of glueRED in L1 and L2 animals when BRC isoforms are ectopically expressed (data not shown). This early expression of glueRED or glueGRN is never observed in control animals or in crosses where EcR-specific isoforms are ectopically expressed. This result may indicate that BRC proteins are sufficient for SGS3 production at any stage of larval salivary gland development, but that critical levels of BRC isoforms are normally restricted to mid-to-late L3 stages in wildtype animals [5].

### EcR Is Required for the Induction of Other Glue Genes

**Figure 5:**
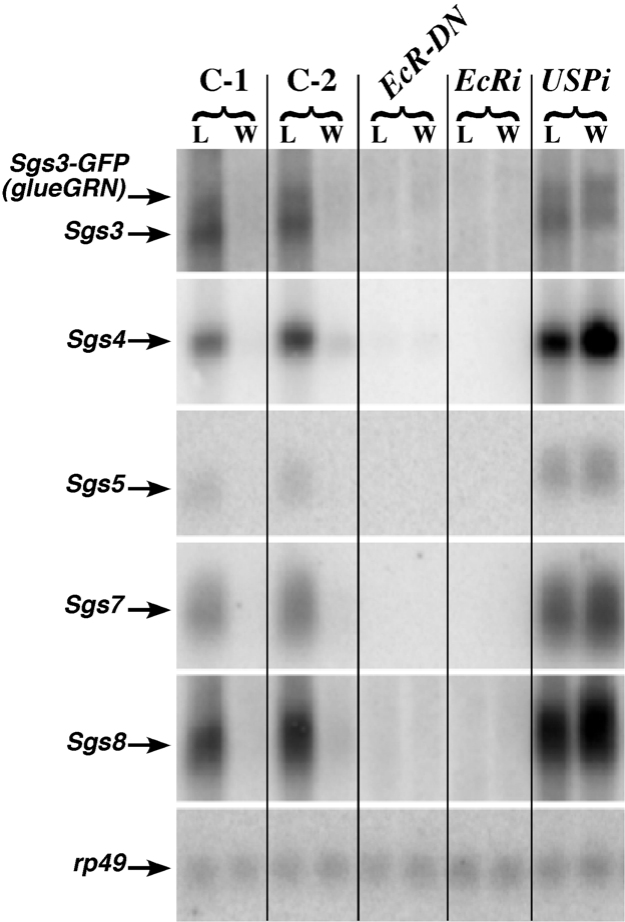
EcR, but Not USP, is Needed for Glue Synthesis. Northern blots were produced from whole-animal extracts at the wandering-L3 (L) and white prepupal (W) stages. Because the glue genes are known to be repressed by the 20E pulse that triggers secretion, RNA levels for each of the 5 different glue genes examined [*Sgs3* (and its derivative *glueGRN*), *Sgs4*, *Sgs5*, *Sgs7*, and *Sgs8*] are expected be high in (L) and low or undetectable in (W) as they are in the controls (C-1 and C-2). C-1 is an extract from the parental *glueGRN* stock and C-2 is an extract from the “driver only” control (*glueGRN* crossed to *sgGal4*). However, when *EcR-DN* or *EcRi* is expressed in the salivary glands, no glue expression can be detected. Blots were hybridized for *rp49* as a loading and transfer control. Interestingly, when glands from the *USPi* cross are assayed, all glue genes examined are expressed, but they are not repressed at the (W) stage. This is the expected result if USP is not required to turn the genes on, but is needed to turn them off at the end of the instar.

Glue is a mixture of at least eight different glycoproteins [41],[42], which are coordinately induced midway through the third instar in a tissue-restricted fashion. To test whether perturbing EcR signaling disrupts the synthesis of most glue proteins, we assayed glue production in EcR-compromised glands in two different ways. First, we examined the glands directly. The cytoplasm of EcR-compromised cells is very small with no detectable secretory granules (Figure 3C,D). If other abundant non-tagged glue proteins were being loaded into granules, this result would not be expected. Second, when we examined the expression pattern of *Sgs3*, *Sgs4*, *Sgs5*, *Sgs7*, and *Sgs8* transcripts by Northern analysis, we found very little signal for any of the five glue genes tested in animals in which EcR was compromised in the salivary gland (Figure 5). Note the normal developmental expression pattern in the control lanes (C-1; C-2). Transcript levels for all glue genes should be high in wandering larvae (L), and they should be low or undetectable at the time of puparium formation (W).

### Induction of the Glue Genes Does Not Require USP

**Figure 6:**
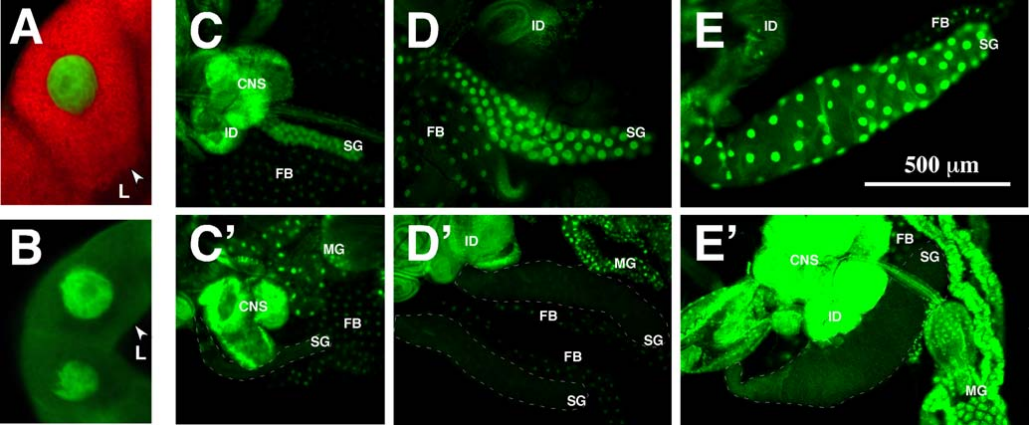
Silencing USP in the Salivary Gland Does Not Block glueRED Synthesis. Confocal images of salivary glands in which RNAi against *USP* is triggered demonstrate that glueRED synthesis is normal but not secreted at the time of puparium formation (A). Overexpressing a wildtype *USP* transgene (*USP+*) produces a synthesis-defective phenotype (B) that is similar to that observed when EcR is compromised. Both images were taken at the same magnification, which is identical to that presented in Figure 3. Confocal images are presented of L3 tissues from wildtype animals (C-E) and larvae in which RNAi was induced against *USP* (*USPi*) in the salivary glands (C′-E′). Tissues are stained with a USP antibody and visualized with a FITC-conjugated secondary. Fluorescence is detected in the nuclei of early-third (C, C′), mid-third (D, D′), and late-third (E, E′) instars. The positions of the salivary glands (SG and dashed outlines), central nervous system (CNS), imaginal discs (ID), fat body (FB), and midgut (MG) are marked for comparison to indicate that *USP* silencing is restricted to the salivary gland as expected with a tissue-specific driver. Note that the gain in E′ is increased to emphasize the lack of USP staining in the nuclei of the salivary glands. Photos C-E′ were taken at the same magnification indicated by the bar in E.

Because all known 20E signaling pathways that control *in-vivo* developmental events are thought to be mediated through an ecdysone receptor consisting of EcR and USP, we wanted to test the requirement for USP in the synthesis of glue. Thus, we utilized a transgenic RNAi construct that contains an inverted repeat of *USP* under *UAS* control (*USPi*). We expressed this construct using the *sgGal4* driver and the reporter genes (*glueRED*; *GFP.nls*) described above in order to selectively silence *USP* in larval salivary glands. Under these circumstances glands were indistinguishable from parental stocks (compare Figure 3A with Figure 6A), and 100% of the glands produced wildtype levels of glueRED (Table 1). This result suggests that USP is not part of the receptor needed for glueRED expression. An alternative explanation is that the *USPi* construct is not effectively knocking down USP levels in the salivary gland, but three lines of evidence make this possibility very unlikely.

**Table 1:** Overexpressing USP blocks the synthesis of glueRED.

Genotype (Transgene Addback)	Empty	Few	Quarter	Half	Full	Number Assayed
***SgGal4 UAS-GFP.nls glueRED UAS-USP*** *i*	0%	0%	0%	0%	100%	50
***SgGal4 UAS-GFP.nls glueRED UAS-USP(+)*** ** (at 25°C)**	34%	2%	17%	25%	22%	56
***SgGal4 UAS-GFP.nls glueRED UAS-USP(+)*** ** (at 29°C)**	53%	11%	16%	13%	7%	61

Glue synthesis was assayed as described in Figure 4.

**Figure 7:**
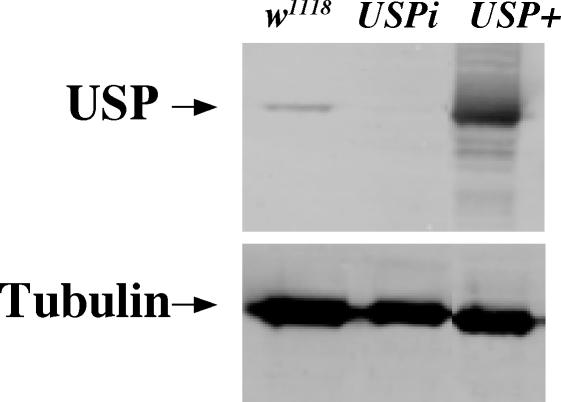
Western-Blot Analysis Comparing USP Levels Derived from Different Transgenic Stocks. Protein extracts were prepared from salivary glands of three different crosses at the wandering-L3 stage. Flies of the tester stock (*sgGal4*; *GFP.nls*; *glueRED*) were crossed to a control to ascertain the normal amount of USP protein present in third-instar glands (*w^1118^*). The same tester stock was crossed to a line in which RNAi against *USP* could be induced in the salivary glands (*USPi*), and to a line in which wildtype *USP* could be overexpressed (*USP+*). The blot was cut, and one part was incubated with antibody against USP and the other was incubated with α-Tubulin as a loading/blotting control.

First, we examined wildtype- and *USPi*-compromised salivary glands for USP protein using a well-characterized USP antibody. As shown in Figure 6, no USP protein can be detected in the nuclei of salivary glands in which *USPi* is expressed. This is in contrast to the wildtype glands of similar L3 stages (compare the tissues marked as SG in C-E with those outlined by a dashed line in C′-E′), and in contrast to *USPi* animals where the fat body (FB), central nervous system (CNS), imaginal discs (ID) and midgut (MG) clearly display the expected nuclear staining. This result is consistent with *sgGal4* driving *USPi* only in salivary glands and not in other tissues. In addition, no USP protein is detected in salivary-gland extracts when a Western-blot analysis is performed on glands expressing the *USPi* construct (Figure 7).

Second, because glue secretion (dumping of granules into the lumen of the gland) at the end of L3 has been shown to be 20E dependent [12] and to require functional EcR and USP [13], we expected that *USPi* glands would not be able to secrete the glueRED that was produced at an earlier stage. This prediction is always supported by data. Note that the photograph of the gland in Figure 6A was taken at the time of puparium formation and that no glueRED can be detected in the lumen (L) of the tissue. In wildtype parental glands, secretion of the tagged glue into the lumen (Figure 3B) always occurs by the white prepupal stage.

Third, because it has been reported that USP is necessary to repress the glue genes at the time of puparium formation, we expect that transcript accumulation for each *Sgs* gene should not decrease at the white prepupal stage. The data presented in Figure 5 (compare L with W in the *USPi* lanes) support this hypothesis.

Another possible caveat for the observation that RNAi against *USP* does not prevent glueRED expression is that a small amount of USP protein may be very stable in the salivary gland and thus not subject to efficient silencing by the RNAi mechanism. Following this logic, the protein turn over might take 4 days to reach a critical threshold level. Thus, there would be enough USP protein for glueRED synthesis in 3-day old larvae (the age when glue genes are induced by 20E), but not enough in 4-day old larvae (the age when 20E causes glue secretion). To test the ability of the *USPi* construct to silence *USP* effectively in a short time frame, we used the *glueGal4* driver (FBst0006870) to express transgenes in the salivary gland from mid-L3 until puparium formation. Under these circumstances glue secretion was blocked even though the *USPi* responder was only being expressed for 24 hours prior to the assay (data not shown).

Because it is known that USP can heterodimerize with EcR at the end of the larval period, we predicted that an overproduction of USP at mid-L3 might prevent a critical amount of EcR from forming the functional receptor needed for glue-gene induction. However, if even a small amount of a receptor consisting of EcR and USP is required to induce the glue genes, overproducing the USP component at an earlier time should not affect the response. Thus, we generated transgenic flies in which the coding information for wildtype *USP* was placed under *UAS* controlling elements. When this transgene (*USP+*) was driven by *sgGal4*, a large amount of USP protein was detected on Western blots of salivary glands (Figure 7), and the production of glueRED was reduced (Figure 6B; Table 1). We verified that this construct produces functional protein by crossing it to flies carrying both the *glueGal4* driver and *USPi* responder. Under these conditions the *USP+* construct was able to rescue the block in glue secretion caused by *USPi*.

**Figure 8:**
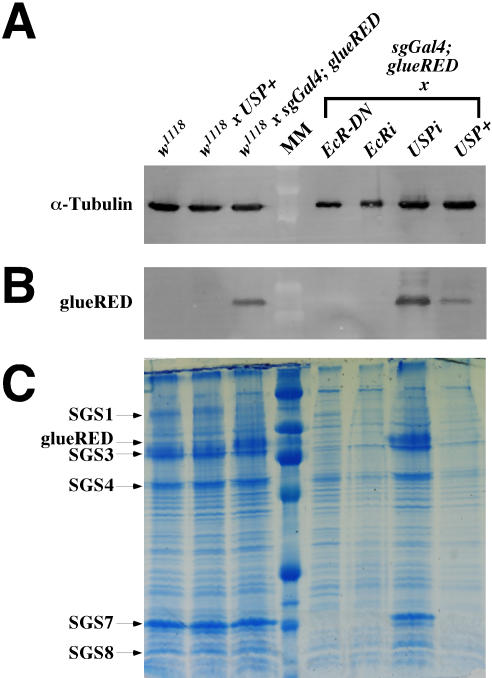
Overexpressing USP Blocks the Synthesis of Glue Proteins. Protein extracts were prepared from salivary glands of seven different crosses at the wandering-L3 stage. Two SDS-PAGE gels were produced, one was fixed and stained (C) and the other was blotted. The blot was cut, and one section was incubated with the Tubulin loading control (A), while the other was incubated with an antibody against DsRED (B). The first three crosses serve as controls for protein levels in the parental stock (*w^1118^*), the “responder only” cross (*w^1118^ x USP+*), and the “tester only” cross (*w^1118^ x sgGal4*; *glueRED*). The remaining 4 crosses were the tester stock (*sgGal4*; *glueRED*) crossed to *EcR-DN*, *EcRi*, *USPi*, and *USP+*. Note that all glue-protein bands from the stained gel are reduced by the *EcR-DN*, *EcRi*, and *USP+* reagents (but not *USPi*) as predicted by the model that glue-genes are induced by 20E through a receptor that requires EcR but not USP. The position of the major glue proteins (arrows) correspond to band sizes observed when secreted glue plugs were precipitated with ethanol and subjected to SDS/PAGE.

Although the overproduction of USP in the salivary gland perturbs glueRED expression (34% of the glands produce no product), the block was not complete because animals were able to express varying levels of glueRED in some salivary-gland cells (Table 1). To more precisely quantify the amount of glueRED produced under these conditions, we performed the Western blot presented in Figure 8. As expected, no DsRED-tagged protein can be detected in the lanes in which *EcR-DN* or *EcRi* are expressed in the salivary glands (Figure 8B). In addition, the levels of glueRED are not reduced when *USPi* is expressed in the salivary glands because both control lanes (*w^1118^ x sgGal4*; *glueRED*) and experimental lanes (*USPi x sgGal4*; *glueRED*) contain the same band intensities when quantified and adjusted for protein loading using α-Tubulin (Figure 8A,B). However, the levels of glueRED are reduced 3 fold when USP is overexpressed (*USP+ x sgGal4*; *glueRED*) in the salivary gland compared to the control and *USPi* lanes (Figure 8B).

One explanation for the reduction, but not elimination of glueRED, is that the amount of USP produced under these conditions is at a threshold level needed to antagonize the 20E-signaling pathway mediated by EcR. To test this hypothesis, we crossed the *USP+* line to *sgGal4*; *GFP.nls*; *glueRED* and raised the larvae derived from the cross at two different temperatures (25°C and 29°C). Because temperatures closer to 30°C are reported to produce higher GAL4 activities [43] (probably because GAL4 is a yeast transcription factor), we predicted that larvae raised at 29°C would produce less glueRED (due to the overproduction of more USP that should antagonize 20E receptor formation). As indicated in Table 1, these differences were observed when animals were raised at the two different temperatures (53% of the glands failed to produce any glue when raised at 29°C compared to 34% that failed to produce any glue when raised at 25°C). In addition, we confirmed that raising control animals at 29°C did not perturb glueRED production, and raising experimental animals at the elevated temperature did not cause a non-specific induction of the heat shock promoter in other tissues because GFP.nls was only detected in the nuclei of salivary glands (data not shown).

Finally, to ascertain the role of USP in the production of other glue proteins, we compared the overall pattern of protein synthesis using Coomassie staining of SDS-PAGE. As shown in Figure 8C, the appearance of most of the glue proteins can be identified when whole salivary-gland-protein extracts are stained because the *Sgs* genes are abundantly expressed in this tissue. We were able to confirm the presence of the major glue bands by comparing extracts of secreted glue plugs [6] that were prepared as ethanol precipitates from the lumens of white prepupae (data not shown). As expected, the accumulation of most glue proteins is reduced drastically in glands in which *EcRi* and *EcR-DN* are expressed. Also as expected, they are not reduced when *USPi* is expressed, but they are affected when USP is overproduced.

Taken together these results are very compelling, and they indicate that *USPi* is very efficient at gene silencing in the salivary gland when driven by *sgGal4*. Therefore, USP is not needed for the 20E-mediated induction of the glue genes through the BRC.

## Discussion

### The Glue Genes Are Induced by 20E

Previous reports using mutants that are defective in 20E production or signaling yielded contradictory results concerning the role of 20E in the induction of the glue genes in the salivary gland. Here we demonstrate that a glue-gene reporter derived from the *Sgs3* gene can be induced by 20E in cultured glands dissected from wildtype animals at mid L3. Furthermore, unlike the 20E mediated events that occur at the end of the larval period, the induction of *Sgs3* and other glue genes is mediated by a lower titer of hormone (10^−9^ to 10^−8^ M). This result is consistent with a report of a small titer of 20E that has been detected in a population of synchronized animals two hours prior to the induction of the glue genes [29]. In addition, because the *ecd1^ts^* mutation probably reduces the concentration of 20E in the hemolymph, mutant animals shifted to the non-permissive temperature might still be exposed to enough 20E to induce the *Sgs* genes. We have also shown that the induction of the glue genes occurs as a secondary response to the hormone because the requirement for EcR can be bypassed if *BRC* isoforms are ectopically expressed. This finding is supported by published evidence that some 20E-regulated transcription factors (BRC, E74B) can be induced in cultured organs by a pulse of hormone that is much lower than that produced at the end of the third instar, ∼10^−8^ M versus ∼10^−6^ M [44].

### The Induction of the Glue Genes Requires a Different 20E Receptor

The dogma for the action of 20E during *Drosophila* development is that EcR and USP are associated as a heterodimer and often bound to the EcREs of target genes. When not bound by ligand, the heterodimer associates with a repressor complex to prevent transcription from those genes. Hormone binding (to the ligand-binding domain of EcR) leads to a conformational change in the complex, the dissociation of the repressor complex, and the recruitment of co-activators for high-level transcriptional activation [reviewed in 19]. Although this model is well supported by evidence that both EcR and USP are required to initiate events during the late-larval and prepupal periods, our study presents compelling evidence for the existence of another *bona fide* receptor for 20E that consists of EcR but does not use USP as its heterodimeric partner.

We have provided evidence that SGS3 production (and probably glue synthesis in general) is a 20E-mediated event. We have also demonstrated that EcR is required for the induction of the glue genes, and that any isoform of EcR can be involved in the activation of *Sgs3*. This result is interesting because EcR-B1 is reported to be the predominant form that is normally expressed in the larval salivary gland [45]. Also, because expression of *BRC* is necessary and sufficient for the induction of *Sgs3*, these experiments suggest that the A/B domain of EcR does not participate in the expression of *BRC* by the smaller pulse of 20E that occurs midway through the L3 stage.

In contrast to the results for EcR, we have provided convincing evidence that USP is not the other half of the heterodimer needed for the 20E-mediated initiation of glue synthesis. In a previous report [13] we confirmed that *USP* mutants can be rescued from embryonic lethality by providing exogenous USP from a heat-shock driven transgene [46]. Furthermore, if these animals are not provided with a source of USP during the L2 and L3 stages (by being deprived of subsequent heat pulses that would induce the transgenic cDNA), they will not pupariate, but they will grow, molt, and express an *Sgs3* derived reporter [13].

In the current study we have used strong tissue-specific drivers that are exclusively expressed in the salivary gland at two different time points. We have demonstrated that the *USPi* stock is an effective reagent for silencing endogenous *USP* in the salivary gland (Figures 6; 7), even if it is only produced for 24 hours before the assay (i. e. inducing it with *glueGal4* blocks glue secretion). Thus, when it is driven during all larval stages (3 days before glue synthesis) no USP protein can be detected by immunostaining, and this absence of USP protein has no effect on the production of glue. To further confirm that USP is not needed for glue synthesis, we demonstrated that when wildtype USP is overexpressed in the salivary gland during the larval stages, glue protein production is drastically reduced. Because USP is known to heterodimerize with EcR at a later developmental stage, the simplest explanation for this observation is that extra USP protein is preventing EcR from forming the functional 20E receptor needed for glue synthesis in mid L3. Such a result is not expected if only a small amount of functional EcR/USP is needed to induce the glue genes.

Interestingly, other researchers have observed similar effects. One report generated clones of *usp-/usp-* mutant tissue in the salivary gland, and although they do not discuss the effects of glue production in mutant tissue, the presence of glue granules is apparent in the clones from late-L3 glands [47]. This and other studies also describe the developmental differences of clones of *usp-* tissue in imaginal discs.

For example, movement of the morphogenetic furrow—a 20E mediated event responsible for eye development [48]—is actually accelerated across a *usp-* patch of tissue [49],[50]. In addition, others have noted that the 20E dependent differentiation of chemosensory neurons in the wing margin occurs precociously in the absence of USP function [51]. Furthermore, when target-gene expression is examined, transcripts from the *BRC* (*BRC-Z1*) accumulate earlier in development in mutant clones within the eye and wing discs [47],[51]. These observations led to the hypothesis that in the absence of ligand, the EcR/USP heterodimer can act as a repressor in some tissues by binding to the response elements of a select group of target genes. The function of the hormone is to de-repress the target genes by removing the EcR/USP complex from the promoter region allowing other bound transcription factors to activate transcription [34]. Thus in a *usp-* clone, genes controlled by this mechanism should be precociously activated. We do not think that the induction of the glue genes is controlled by a de-repression of *BRC* through EcR/USP for two reasons. First, the glue genes are not induced (de-repressed) if *EcR* is silenced with an *EcRi* construct. Second, we do not see precocious activation of glue genes when a *USPi* construct is expressed.

Our model proposes that USP is acting as a repressor by heterodimerizing with EcR to prevent the association of EcR with another nuclear-hormone receptor (NR-X). Our hypothesis may also explain some of the data generated with the use of *usp-* clones in imaginal discs. For example, if we assume that movement of the morphogenetic furrow is induced by an earlier and lower pulse of 20E (as has been reported for *Manduca*) [52], we would speculate that furrow movement is controlled by EcR/NR-X regulating downstream genes including *BRC-Z1*. The normal presence of USP in this tissue at that time might serve to control the amount of functional EcR/NR-X available for high-affinity hormone binding. Thus in a *usp-* clone, we would expect the morphogenetic furrow to move faster over the patch and the induction of *BRC-Z1* to be premature. Such observations were reported [47],[49],[50].

The normal expression of USP in the salivary gland at mid L3 (Figures 6; 7) may also be needed to ensure that the response of glue-gene induction is precisely regulated. In any case, the induction of a 20E regulated pathway that does not require USP as part of the receptor has no precedence in the *Drosophila* literature. Thus, a better characterization of this response at the molecular level is critical for our understanding of normal insect development.

### Transcriptional Regulation of the Glue Genes

In this report we demonstrate that EcR is necessary for the expression of most of the glue genes at mid L3, and that USP is not needed for this expression. In addition, we show that any isoform of BRC can be sufficient for *Sgs3* transgene expression even if the EcR component of the receptor is compromised with EcR-DN, and that overexpression of some BRC isoforms in first- and second-instar larvae is enough to induce expression of the *Sgs3* transgenes days before they would normally be transcriptionally active.

However, it is interesting to note that although *Sgs3* and *Sgs4* appear to be coordinately expressed in mid-L3 salivary glands, different binding sites for regulatory proteins have been identified in their promoter/enhancer regions. These include response elements for EcR/USP, and binding sites for BRC [40], GEBF-I (FBgn0013970) [53], Forkhead (FBgn0000659) [54]–[56], and SEBP3 (FBgn0015293) [57]. The binding of different transcription factors to these sites may modulate the levels of expression of the two genes or they may contribute to their restricted expression patterns in the salivary gland or other tissues. For example, although we have shown that *Sgs3* derived transgenes are exquisitely restricted to the salivary glands of third-instar larvae, others have reported the expression of different glue genes in tissues outside this cell type. These include *Sgs4* expression in the proventriculus [58] and *I71-7* expression in the midgut and hemocytes [59]. Such expression patterns raise the interesting possibility that these highly glycosylated mucin secretions may perform other functions stemming from their propensity to form a sticky substance in aqueous solution. These functions could include the formation of the peritropic membrane around the food or the formation of extracellular aggregates that might be involved in antimicrobial responses [59].

### What Is the Composition of the 20E Receptor Responsible for Inducing the Glue Genes?

If we assume that members of the nuclear-hormone receptor superfamily form dimers to produce the active receptor needed for glue-gene expression, we can formulate two hypotheses concerning the composition of that functional receptor. First, the active receptor may be a homodimer of EcR proteins. Homodimers are known to function as receptors for steroid hormones in vertebrates using a different mechanism of ligand activation than that observed with RXR heterodimeric receptors (USP is the insect homolog of RXR), but to our knowledge no biological activity has been ascribed to EcR homodimers during *Drosophila* development. Our analysis does not rule out the possibility that EcR homodimers are responsible for the induction of the glue genes.

The second possibility is that another member of the superfamily may be able to complex with EcR to transmit the hormone signal. Many of these receptors have pre-existing mutations and many more have *UAS-RNAi* lines that are now available from the RNAi Stock Centers in Vienna (http://www.vdrc.at) and Japan (http://www.shigen.nig.ac.jp/fly/nigfly/index.jsp). At this point we have assayed production of glueRED in mutants or RNAi lines that knock down *DHR38* (FBgn0014859) and *DHR78* (FBgn0015239), but no effects on glueRED synthesis were observed (A. Andres, unpublished observations). However, the existence of transgenic RNAi lines should simplify the analysis because it is expected that when a specific nuclear receptor is silenced in the salivary gland, it should display a phenotype that is defective in glue synthesis. It would then be very interesting to screen the controlling region of the *BRC* to establish the nature of the EcRE(s) that control the response at the molecular level, and to test if this type of receptor could control other developmental events (perhaps molting of the instars or some aspect of early imaginal disc development) that are regulated by 20E during earlier larval stages.

## Materials and Methods

### *Drosophila* Stocks and Culture

All flies were raised on standard cornmeal-molasses medium supplemented with live baker's yeast as recommended by the Bloomington Stock Center (Bloomington, Indiana, United States) (http://flystocks.bio.indiana.edu/Fly_Work/media-recipes/bloomfood.htm). *w^1118^* (FBst0307124), *GFP.nls* [*{UAS-GFP.nls}14* (FBst0004775)], *EcRi* [*{UAS-EcR-RNAi}104* (FBst0009327)], *EcR-DN* [*{UAS-EcR.B1-*Δ*C655.F645A}TP1* (FBst0006869)], and the *EcR* isoform stocks [*EcR-A {UAS-EcR.A}3a* (FBst0006470), *EcR-B1 {UAS-EcR.B1}3b* (FBst0006469), *EcR-B2 {UAS-EcR.B2}3a* (FBst0006468), and *EcR-C {UAS-EcR.C}Tp1-4* (FBst0006868)] were obtained from the Bloomington Stock Center.

The following stocks were provided as generous gifts: *UAS-hid* [60] from Eric Baehrecke, the *hsGal4* driver on the third chromosome [20] from Robert Holmgren, and the stocks containing specific isoforms of the *BRC* (*UAS-BRC-Z1*, *UAS-BRC-Z2*, *UAS-BRC-Z3*, and *UAS-BRC-Z4*) [61] from Xiaofeng Zhou.

### Generation of Transgenic Flies

Transgenic flies containing *glueRED* were prepared by digesting *pDsRed2-C1* (Clonetech, Palo Alto, California, United States) with *AgeI* and *KpnI* restriction enzymes to isolate a DNA fragment containing the open reading frame for *DsRED*. This fragment was cloned into *pBS-Sgs*Δ*3GFP* [13] that was digested with the same enzymes to remove the *eGFP* tag and generate a vector with compatible ends. The resulting intermediate construct was digested with *AgeI* and the 3′ recessed ends were filled in and religated to restore the open reading frame between *Sgs3* and *DsRED*. The *Sgs3-DsRED* sequence was removed from the Bluescript vector (Stratagene, La Jolla, California, United States) as a *NotI/KpnI* fragment and inserted into the *NotI* and *KpnI* sites of the *pCaSpeR-4* fly transformation vector (FBmc0000178). DNA was sent to the vonKalm laboratory at the University of Central Florida for the generation of transgenic flies using standard techniques [62].

To produce the *UAS-USPi* stock, a PCR fragment was amplified from a *USP* cDNA plasmid [63] using the primers AAGAATTCGGTACCAGTATCCGCCTAACCATCC and TTAGATCTCGCTTCATCTTTACACTCAG. The resulting amplification product (corresponding to a 924 bp fragment between positions 467 and 1390 relative to the *USP* mRNA sequence) was cloned in the *pUAST* vector (FBmc0000383) using two steps. First a reverse fragment was placed between the vector *BglII* and *KpnI* sites. A second forward-orientated fragment was cloned between *EcoRI* and *BglII* sites. Recombinant *UAS-USPi* constructs were transformed at 30°C in Sure-competent bacteria (Stratagene) to minimize DNA recombination and screened using appropriate restriction enzyme digestions. Transgenic lines were generated as previously described using a *w^1118^* strain as a recipient stock.

*UAS-USP+* stocks were prepared as follows: The vector *pUAST-USP+* was constructed by PCR amplification of the *USP* open reading frame with the forward primer TTTTGCGGCCGCACC ATG GAC AAC TGC GAC CAG GAC and the reverse primer TTTTTCTAGA CTA CTC CAG TTT CAT CGC CAG using *pZ7-1* cDNA as a template [63]. The *NotI* and *XbaI* restriction sites flanking the PCR product were used for subsequent ligation into the corresponding sites in the *pUAST* vector. The *pUAST-USP+* vector was transformed into flies at the Duke University Medical Center.

The *UAS-Control* line (*LA1216*) contains an insert of the construct *P{Mae-UAS.6.11}* (FBtp0001327). This vector was designed for gene-mis-expression screens because it contains a copy of the UAS/GAL4 binding sequences oriented to express flanking genes when inserted into the genome [64].

### Selecting a Salivary-Gland Specific Driver

We tested four *Gal4*-drivers obtained from the Bloomington Stock Center [*AB1-Gal4* (FBst0001824), *C147-Gal4* (FBti0024396), *T155-Gal4* (FBti0002598), and *34B-Gal4* (FBst0001967)] with expression patterns reported to be restricted to the larval salivary gland. To ascertain which of these was best for tissue-specific expression studies, we crossed them to a stock in which the *hid/Wrinkled* cell-death gene (FBgn0003997) was expressed under *UAS* control. Because the major function of the salivary gland in the larval stages is the reported synthesis of mucin-like proteins that help lubricate the food as it moves through the gut [2],[3], we reasoned that animals could survive without a salivary gland only if they were provided a diet of freshly produced moist yeast paste. Thus, by using *UAS-hid* we could ablate the salivary gland and test if such animals were viable when raised on soft food.

The initial analysis using the above listed drivers indicated that no larvae were able to survive, probably due to expression of the *hid* gene in other vital tissues. But because these are derived from the *GawB* vector, we used a *heat shock 70-Gal4* driver that is also *GawB* derived. These animals were able to survive to puparium formation when crossed to *UAS-hid* and raised on a diet of freshly prepared yeast paste. Crossing *hsGal4* to a stock containing both *UAS-hid* and *UAS-GFP.nls* confirmed that larval salivary glands could not be detected and were ablated. Great care was exercised to raise the animals crossed to *sgGal4* at temperatures below 30°C to prevent exposing them to a stress that might induce *Gal4* in all cells. Because the *GFP.nls* transgene was used in most of the experiments, a non-specific response could easily be detected by the presence of green nuclei in other tissues.

### Salivary Gland Organ Culture

20E (Sigma, St. Louis, Missouri, United States) was prepared as a stock solution of 10^−2^ M in 100% ethanol and stored at −20°C. The stock solution was diluted to the proper working concentration in Schneider's medium (Sigma).

Flies of the appropriate genotype were crossed and reared in a small population cage containing approximately 500 females and 500 males. The cage was presented with hard-agar plates (10% molasses, 3.5% agar) containing a dab of fresh yeast paste (prepared as a 1∶1 mixture of dry baker's yeast with water) 2–3 times per day to collect fertilized eggs. Collection plates were aged at 25°C and first-instar larvae were collected in 1-hour intervals as they hatched. The first-instar larvae were added in groups of 100 to vials containing standard cornmeal-molasses-yeast medium and aged at 25°C for approximately 68 hours (a developmental stage that precedes glue induction by approximately 4 hours) before being washed from the food with Schneider's medium. Animals were torn in half lengthwise using small dissecting forceps (Fine Scientific Tools, Foster City, California, United States). Larvae prepared in this manner were transferred to clean microscope slides containing 25 µl of Schneider's medium with or without 20E. A range of 20E dilutions (10^−6^, 10^−7^, 10^−8^, 10^−9^ M) was prepared for each experiment. Small strips of Number 1 Whatman filter paper (Millipore, Billerica, Massachusetts, United States) were placed around the culture as spacers before adding a 22 mm^2^ coverslip. The culture was placed on a platform shaker in a box into which O_2_ was continuously infused during the culture period. Cultures were incubated at 25°C for 4–6 hours before being assayed for glue production as detected by the presence of green fluorescent protein from the *glueGRN* transgene.

### Microscopy and Imaging

Whole larvae were selected from the food, washed 3× in water, blotted on filter paper, placed in a depression slide, and killed with a few drops of ether. After the ether evaporated, animals were mounted in glycerol between two slides using glass coverslips as spacers. Larvae were photographed within 30 minutes of preparation. For isolated tissues, animals were dissected in *Drosophila* PBS (DPBS) [65] or Schneider's medium. Low-resolution images of whole animals or dissected tissues were obtained on a Leika fluorescent stereo microscope containing filter cubes for GFP and/or DsRED. Images were captured with the Spot Insight QE Model #4.2 digital camera (McBain Instruments, Chatsworth, California, United States) and prepared with Canvas (ACD Systems, Miami, Florida, United States) graphics software.

High-resolution images of dissected salivary glands were imaged on a LSM 510 Axioplan confocal microscope (Carl Zeiss SMT, Peabody, Massachusetts, United States) equipped with LSM 510 image-analysis software.

### Northern Blots

Northern blots were prepared as previously described [5]. Briefly, RNA was isolated from larvae by grinding animals in SDS lysis buffer, digesting the homogenate with 250 μM Proteinase K (NEB, Ipswich, Massachusetts, United States), extracting the sample with phenol/chloroform, and precipitating the aqueous phase with ethanol. Ten micrograms of total RNA were fractionated on 1% formaldehyde/MOPS/agarose gels and blotted onto Duralon-UV membranes (Stratagene). Probes for each *glue* gene and the *rp49* control were prepared as gel-isolated fragments from digested clones and hybridized with labeled random oligonucleotides using a Prime-it kit (Strategene) and ^32^P dCTP (GE Healthcare, Piscataway, New Jersey, United States) as previously described [5]. After washing, signals were detected using the Typhoon 8600 Variable Mode Phosphorimager equipped with Image Quant scanning software (GE Healthcare).

### Protein Detection

Dissected tissues were prepared for antibody staining as previously described [66]. Tissues were stained using the AB11 USP mouse monoclonal antibody [67] (gift from Carl Thummel) at a dilution of 1∶50. Protein levels were visualized using a goat-anti-mouse secondary antibody conjugated to FITC (Jackson Immuno Research, West Grove, Pennsylvania, United States).

To prepare protein extracts for Coomassie staining or Western-blot analysis, salivary glands were dissected in DPBS as described above. Typically 10–20 pairs of glands were collected in DPBS, pelleted in a microfuge, and resuspended in lysis buffer containing a cocktail of protease inhibitors [68]. Glands were homogenized and boiled for 5 minutes before being stored at −20°C for less than one week. Samples were divided in two and resolved on separate 12% SDS polyacrylamide gels that were run in the same electrophoresis rig. One was stained with Coomassie brilliant blue (J. T. Baker, Phillipsburg, New Jersey, United States) and the other was transferred to Immobilon P membranes (Millipore) as previously described [66]. Blots were incubated with the following antibodies: mouse anti-α-Tubulin primary (Sigma) diluted 1∶15,000; rabbit anti-DsRED primary (Clontech) diluted 1∶15,000; mouse anti-USP primary diluted 1∶100; goat anti-mouse-HRP secondary (Jackson Immuno Research) diluted 1∶40,000; and goat anti-rabbit-HRP secondary (Jackson Immuno Research) diluted 1∶25,000.

Protein levels were visualized and quantified using Chemi-luminescence ECL(+) Western-blotting detection system (GE Healthcare) and a Typhoon 8600 Variable Mode Phosphorimager (GE Healthcare).

### Accession Numbers

The FlyBase (http://flybase.bio.indiana.edu/search/) identification numbers are used in this work to describe genes, gene products, vectors, and *Drosophila* stocks.
